# Differential Analysis of Ovarian and Endometrial Cancers Identifies a Methylator Phenotype

**DOI:** 10.1371/journal.pone.0032941

**Published:** 2012-03-05

**Authors:** Diana L. Kolbe, Julie A. DeLoia, Patricia Porter-Gill, Mary Strange, Hanna M. Petrykowska, Alfred Guirguis, Thomas C. Krivak, Lawrence C. Brody, Laura Elnitski

**Affiliations:** 1 DIR/GTB Genomic Functional Analysis Section, National Human Genome Research Institute, National Institutes of Health, Bethesda, Maryland, United States of America; 2 DIR/GTB Molecular Pathogenesis Section, National Human Genome Research Institute, National Institutes of Health, Bethesda, Maryland, United States of America; 3 Department of Obstetrics, Gynecology and Reproductive Sciences, University of Pittsburgh Medical School, Pittsburgh, Pennsylvania, United States of America; 4 School of Public Health and Health Services, George Washington University, Washington DC, United States of America; 5 Department of Obstetrics and Gynecology, Rush University, Chicago, Illinois, United States of America; John Hopkins Medical School, United States of America

## Abstract

Despite improved outcomes in the past 30 years, less than half of all women diagnosed with epithelial ovarian cancer live five years beyond their diagnosis. Although typically treated as a single disease, epithelial ovarian cancer includes several distinct histological subtypes, such as papillary serous and endometrioid carcinomas. To address whether the morphological differences seen in these carcinomas represent distinct characteristics at the molecular level we analyzed DNA methylation patterns in 11 papillary serous tumors, 9 endometrioid ovarian tumors, 4 normal fallopian tube samples and 6 normal endometrial tissues, plus 8 normal fallopian tube and 4 serous samples from TCGA. For comparison within the endometrioid subtype we added 6 primary uterine endometrioid tumors and 5 endometrioid metastases from uterus to ovary. Data was obtained from 27,578 CpG dinucleotides occurring in or near promoter regions of 14,495 genes. We identified 36 locations with significant increases or decreases in methylation in comparisons of serous tumors and normal fallopian tube samples. Moreover, unsupervised clustering techniques applied to all samples showed three major profiles comprising mostly normal samples, serous tumors, and endometrioid tumors including ovarian, uterine and metastatic origins. The clustering analysis identified 60 differentially methylated sites between the serous group and the normal group. An unrelated set of 25 serous tumors validated the reproducibility of the methylation patterns. In contrast, >1,000 genes were differentially methylated between endometrioid tumors and normal samples. This finding is consistent with a generalized regulatory disruption caused by a methylator phenotype. Through DNA methylation analyses we have identified genes with known roles in ovarian carcinoma etiology, whereas pathway analyses provided biological insight to the role of novel genes. Our finding of differences between serous and endometrioid ovarian tumors indicates that intervention strategies could be developed to specifically address subtypes of epithelial ovarian cancer.

## Introduction

Ovarian cancer has an incidence of 21,500 cases per year in the United States and 204,000 worldwide, with an estimated annual mortality of 125,000 women. The condition ranks as the 5^th^ leading cause of cancer-related deaths for women in the United States; the high mortality rate is a consequence of the asymptomatic nature of early-stage disease and the absence of a reliable screening test. The majority of cases (75%) are diagnosed at an advanced stage (III or IV) wherein the 5-year survival rate is less than 30% [1].

Of the four major histopathologic subtypes, serous is the most common, followed by endometrioid, mucinous and clear cell types. These subtypes have distinctive gene expression profiles [2] and are classified by virtue of their morphologic resemblance to normal fallopian tube, endometrium, endocervix and endometrial clear cells, respectively [3]. The resemblance between tumor subtypes and distant tissues is consistent with models that propose migration of precursor lesions from disparate origins, such as the fallopian tube [4] or the mesothelial covering of the peritoneal cavity [5]. For this reason, ovarian serous tumors (which resemble Mullerian epithelia) can be legitimately compared to normal fallopian tube (which is derived from Mullerian epithelia). Nevertheless, the ovarian surface epithelial (OSE) layer [6] shares its origin with epithelia of the endometrium (known as celomic epithelium [7]) and remains a plausible alternative explanation for “de novo” tumorigenesis.

Like serous tumors, the origin of endometrioid tumors is controversial [8], and progenitor cells have been proposed to originate from non-ovarian sources, such as endometriosis [9]. Tumors with endometrioid histopathology are diagnosed in both the uterus and ovary. They frequently co-occur, as synchronous primary tumors or metastases from uterus to ovary [10]. Whereas molecular differences have been reported for dual primary tumors, metastatic tumors are clonally identical [11].

Gene expression studies of ovarian tumors intended to detect cancer-specific profiles have yielded modest success and limited reproducibility [12], [13], [14], [15]. However, mutational profiling studies have yielded more consistent results, showing that both serous and endometrioid tumors have aggressive, high-grade subtypes [16], [17], [18] with mutations in the *TP53* gene [19], [20], [21] despite their obvious histological differences [16], [17], [18]. Low-grade subtypes are most common in endometrioid tumors with mutations predominantly occurring in *WNT* and *PIK3CA* pathways [2].

In concert with gene expression and mutational profiles, delineating the epigenome of tumor cells should reveal relationships among samples reflecting common embryological origins, similar histopathological outcomes, or shared mutational events. In ovarian tumors, DNA methylation silences expression of critical genes [22], [23], and creates genetic haploinsufficiency [24], while hypomethylation at other sites enables expression of normally silenced genes. As proof of principle, site-specific patterns of DNA methylation were recently used to distinguish four subtypes of epithelial ovarian cancers, using a total of 1,505 target CpG loci [25], [26].

We hypothesized that DNA methylation patterns in ovarian tumors would resemble cells from their putative tissue of origin, with a small number of changes representing events associated with malignancy, that uniquely represent each tumor subtype. Moreover, we also hypothesized that uterine and ovarian endometrioid tumors were related by pathogenic mechanisms, which would be observed in DNA methylation patterns. To address these ideas, we examined methylation profiles of 27,578 target CpG sites representing 14,495 genes in the human genome, using DNA derived from serous and endometrioid ovarian tumors, normal fallopian tube and normal endometrium, and primary and metastatic endometrioid endometrial tumors. This large dataset was analyzed using a supervised analysis followed by *de novo* classification using unsupervised computational clustering. To improve the strength of the epigenetic profiling technique, we included raw methylation data from serous tumors and normal fallopian tube generated through The Cancer Genome Atlas (TCGA). These samples were analyzed using the same methylation platform, and performed by independent research laboratories using independent tumor specimens.

## Results

### Experimental assay and design

We analyzed the DNA methylation status of genomic samples using the Illumina Infinium platform. DNA was treated with bisulfite to convert unmethylated cytosines to uracil, leaving methylated cytosines unchanged. The hybridization reaction on the HumanMethylation27 Illumina BeadChip provided signal specific to the methylated and unmethylated states, using the Illumina single base extension assay protocol [27]. The differential hybridization of probes to methylated and unmethylated target sites was tabulated as the fraction of the total signal that corresponded to the methylated state. The initial sample set represented various tissue and tumor types including normal fallopian tube, normal endometrium, ovarian papillary serous carcinoma, ovarian endometrioid carcinoma, and primary and metastatic endometrial endometrioid carcinoma from 42 patients (Table 1). Technical replicates indicated highly reproducible results for the assay (Figure S1). In addition, we included data from the same Illumina methylation platform for 12 additional samples from the public database of The Cancer Genome Atlas project (TCGA). This set comprised 8 control samples (normal fallopian tube) and 4 tumor samples (ovarian serous), contributed in a single batch of samples examined under consistent experimental conditions from one data provider.

**Table 1:** Experimental samples for data clustering and validation testing.

Sample Type	Non-cancerous	Stage1	Stage2	Stage3–4
Fallopian tube	4			
Endometrium	6			
Serous tumor		3	4	5
Ovarian endometrioid tumor		5	2	2
Endometrial endometrioid tumor, validation set		6		
Ovarian metastases of endometrial tumor				5
TCGA Fallopian tube normal	8			
TCGA serous tumor				4
Serous tumor, validation set				25

### Bulk methylation

**Figure 1:**
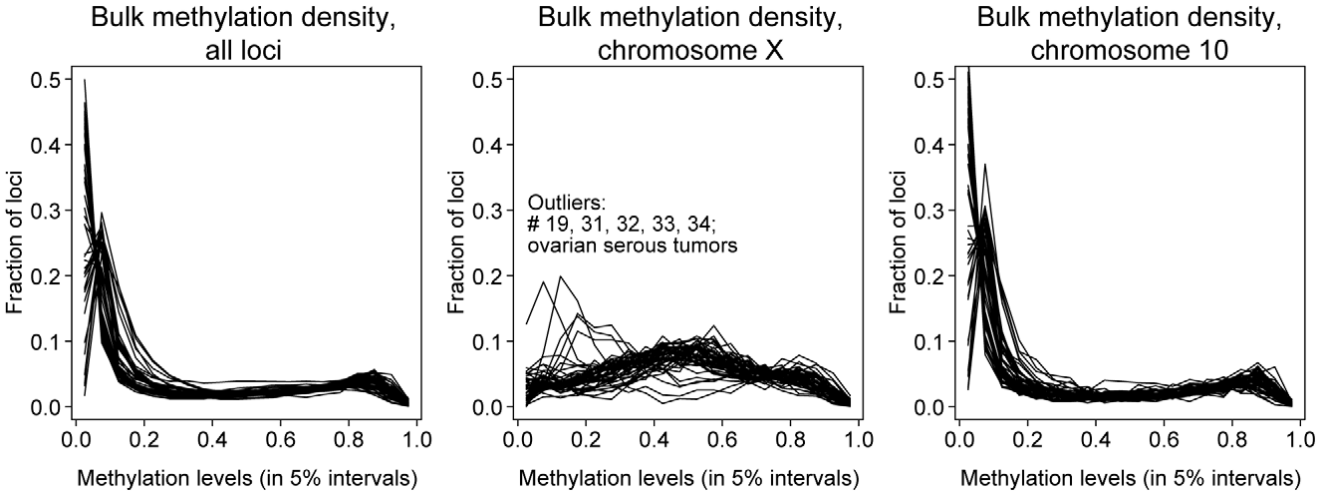
Bulk methylation levels across genomic loci. Frequency of methylation at all loci for a given level of methylation (range 0 to 1). Each biological sample is represented as a single line; all non-metastatic samples were plotted. Respectively, panels from left to right contain all loci (N = 27,561), X chromosome only (N = 1038), or chromosome 10 only (N = 1044).

To assess gross changes in degree of methylation, we examined aggregate methylation levels of all samples. Assay values are reported as the proportion of fluorescence arising from the probe for the methylated state, from 0 (all DNA unmethylated) to 1 (all DNA methylated). Comparing all samples, the vast majority showed consistent methylation profiles. Across the genome, most assayed sites had methylation levels between 0 and 0.2, small numbers of sites had levels between 0.2 and 0.8, and a slightly larger number had levels of 0.8 to 1 (Figure 1).

In contrast, considering only the X-chromosome, single-copy silencing by random X-inactivation was expected to produce a methylation level of 0.5. The observed pattern showed a broad peak centered at 0.5 for most samples, even though the tumor samples had more extensive heterogeneity. Five serous tumor samples showed a distinct profile, with many loci being unmethylated (i.e., methylation level <0.2), indicating either a failure to maintain X-inactivation or copy number alterations with relative excess of the active X [28]. To assess whether this observed variation was simply due to the small number of loci on the X-chromosome, we also considered methylation levels on chromosome 10, which had a similar number of probes. The pattern for chromosome 10 resembled the pattern across all autosomal loci, with high levels of similarity from sample to sample.

### Supervised analysis

We first considered whether the tumor subtypes (defined by histopathology) corresponded to specific methylation profiles. With the inclusion of the TCGA samples, 12 normal fallopian tube samples and 16 ovarian serous tumors yielded sufficient statistical power for a direct comparison. Probes with poor quality control, high variability in the controls, or located on X or Y-chromosomes were not considered (see Methods). Using a Wilcoxon summed-rank test to identify sites that consistently associated with prior classification, 36 were significant at p<0.05 after multiple-testing correction (14 at p<0.01; Table 2). Three genes were identified as members of the canonical pathway for ovarian cancer in an Ingenuity Pathway Analysis (IPA) (Table 2; [29] [30] [31] [32]). Supervised analysis of the ovarian endometrioid tumors against the fallopian tube or endometrial controls was not performed due to small numbers of samples.

**Table 2:** Differential methylation of genes in supervised analysis, p<0.05.

Gene	Chr	Pos	ControlMedian	SerousMedian	P-value	Probe
*HSPA2*	14	64075923	0.25	0.08	0.0016	cg01520924
*CLIC3*	9	139010829	0.18	0.06	0.0016	cg02189785
*CBFB*	16	65620188	0.26	0.10	0.0016	cg06766367
*FAM3C*	7	120823965	0.31	0.17	0.0016	cg14175438
*PIP5K2C*	12	56270986	0.15	0.07	0.0016	cg25133016
*CHAC1*	15	39032145	0.86	0.77	0.0016	cg26065841
*XLF*	2	219733988	0.26	0.16	0.0033	cg04587910
*SPINT2* *	19	43446608	0.14	0.06	0.0033	cg13301014
*SNTB1*	8	121894651	0.83	0.36	0.0033	cg14992108
*LOC387882*	12	104249242	0.13	0.06	0.0033	cg26940261
*PARP3*	3	51951707	0.71	0.43	0.0066	cg12554573
*DSCR6*	21	37299808	0.83	0.91	0.0066	cg12564962
*EIF4E*	4	100070234	0.29	0.10	0.0066	cg15633390
*MRGPRX4*	11	18149936	0.59	0.36	0.0066	cg16446783
*LGP1*	17	37600206	0.25	0.10	0.012	cg08468689
*PDPK1*	16	2527081	0.80	0.44	0.012	cg14444710
*PTGES* *	9	131555630	0.76	0.59	0.012	cg17683775
*MGC4399*	1	9521654	0.82	0.73	0.012	cg18783781
*CD58*	1	116914377	0.27	0.09	0.012	cg21039631
*FLJ00060*	19	59739533	0.80	0.51	0.02	cg03602500
*CDKN3*	14	53933994	0.13	0.05	0.02	cg03724882
*GIT1*	17	24941283	0.31	0.17	0.02	cg05379350
*PLEKHF1*	19	34847706	0.49	0.26	0.02	cg05512099
*DPP8*	15	63597257	0.11	0.04	0.02	cg06993413
*FLJ22555*	2	200527315	0.77	0.56	0.02	cg15761233
*CSRP3*	11	19180211	0.72	0.43	0.02	cg19216731
*HDAC1* *	1	32529881	0.37	0.20	0.02	cg24468890
*HUNK*	21	32166623	0.16	0.07	0.02	cg25048564
*SOCS3*	17	73866797	0.25	0.02	0.02	cg27637521
*ZNF154*	19	62912474	0.09	0.56	0.031	cg08668790
*FGF18* *	5	170778215	0.62	0.47	0.031	cg15699524
*S100A8*	1	151630204	0.44	0.15	0.031	cg24898863
*TLCD1*	17	24076955	0.14	0.05	0.049	cg07195577
*C7orf34*	7	142346962	0.25	0.13	0.049	cg10896774
*NFYB*	12	103056507	0.21	0.12	0.049	cg10954182
*DNAJC14*	12	54509148	0.22	0.12	0.049	cg11380624

* appears in IPA canonical pathway for ovarian cancer.

### Unsupervised clustering

To address whether other sample divisions with shared molecular phenotypes existed and to gain a broader picture of the relationships between the sample types, we moved to unsupervised clustering. Utilizing the complete set of 31 primary tumor samples and 18 normal tissues (and excluding the 5 metastatic samples used for secondary analysis), we limited this analysis to probes that were in the top 500 when ranked by variance, as reported by Houseman et al. [33] to reduce the dimensionality of the data. Results of multiple clustering algorithms converged on the same interpretation of distinct phenotypic groups (Figure S2). K-means clustering and partitioning using a β-mixture model designed for the data from this platform [33] both strongly supported the existence of 3 primary groups, roughly corresponding to control-type samples, serous tumor-type samples, and endometrioid samples. Additional analysis with hierarchical clustering (across multiple distance metrics and linkage methods) strongly supported the control-type and endometrial type clusters, and indicated the serous tumor-type samples were an outgroup from the control samples, but was inconsistent as to whether these samples formed a distinct subgroup (Figure S2).

**Figure 2:**
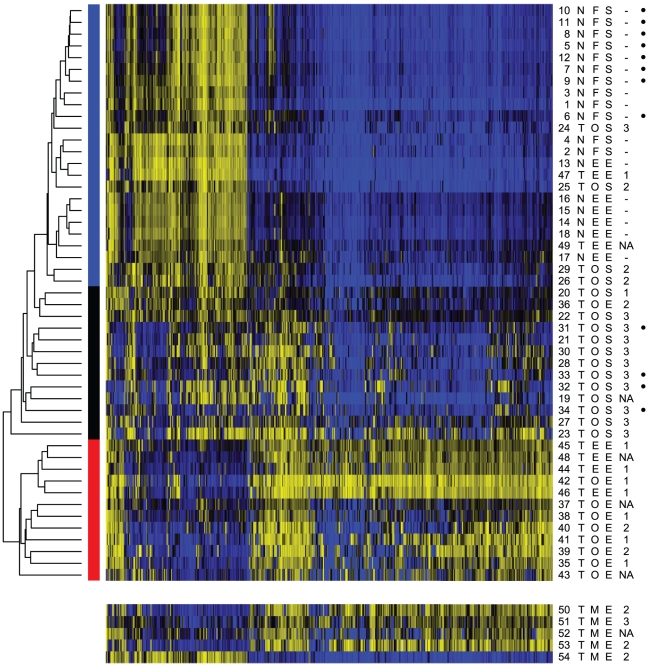
Methylation status at top 500 most variable probes. Heatmap of methylation levels; blue = 0.0, black = 0.5, yellow = 1.0. At left, a representative sample of hierarchical clustering, and color blocks giving the consensus groups. Six columns on the right give sample characteristics: number; tumor (T) or normal (N); location in ovary (O), fallopian tube (F), or endometrium (E); histology of serous (S) or endometrioid (O); grade (1–3; where symbols used are ‘-’ for normals and ‘NA’ for information not available.) and at far right, dots for public TCGA data. Five samples at bottom are ovarian metastases from endometrial endometrioid tumors, excluded from the initial analysis and clustering.

The consensus groupings, as shown in Figure 2, are marked with a colored bar to indicate the normal-type, serous-type or endometrioid-type. Notably, the control group contained normal fallopian tube and normal endometrium, indicating a consistent phenotype across both tissues within the set of featured probes. The TCGA samples clustered with their identified sample types, confirming the reproducibility and robustness of the results as these tumors were obtained and classified at various institutions. Exclusion of the TCGA samples from the analysis had relatively small impact on the results, in which a marked division remained between the endometrioid and serous or control samples; however, only weak support remained for a subdivision of the serous-type tumors from the control samples.

The cluster of endometrioid samples, including tumors from both ovarian and uterine sites, displays a remarkably altered profile, with methylation at numerous sites that are normally unmethylated CpG islands, and a loss of methylation at sites that are normally methylated. The extent and reproducibility of these changes is strongly reminiscent of the methylator phenotypes noted in other cancers [34] [35]. A methylator phenotype has previously been proposed for endometrial endometrioid carcinoma, based on methylation of promoters of a few target genes [36], but has not to our knowledge been described in a genome-level survey or in ovarian cancer.

These data confirm the hypothesis that endometrioid type tumors, whether at ovarian or uterine sites, share similarities at the molecular level. To further address this finding, we analyzed five ovarian metastases derived from primary endometrial tumors using a nested log-likelihood-ratio test. This test addressed consistency of clustering with the primary endometrioid samples versus the combined serous tumors and controls, and secondarily enabled classification within the serous or control groups when necessary. Four of the five samples were strongly identified as endometrioid-type, whereas the fifth was more similar to the control samples (sample 54; Figure 2). The fifth sample does not appear grossly altered from the methylation of normal endometrial tissue, indicating that the majority tumor phenotype is not universal. This outlier may represent an uncommon subdivision of endometrioid tumors, resulting from different underlying pathology, however it does not represent a low-grade tumor (Figure 2).

Assessment of primary tumors of all histopathologies also identified infrequent outliers. Examples included endometrial endometrioid samples 47 and 49, which clustered with normal tissues and contributed to the 15% of samples that showed discordant placement relative to their assigned histopathology (grade 1 and grade not available, respectively; Figure 2). Four ovarian serous tumors also clustered with normal tissues, showing very limited changes in methylation relative to controls (grades 2 and 3). Given the phenotypic similarity of these samples to normal controls, the biological underpinning of this tumor subset requires further investigation. Additionally, one ovarian endometrioid sample grouped with the serous tumors (sample 36, grade 2), suggesting either a rare endometrioid subtype with a more aggressive, serous-like profile, or mistyping of a poorly differentiated sample.

### Differential analysis of clusters

Given the three primary groupings provided by the unsupervised clustering analysis, we wished to identify methylation loci most predictive of membership in a particular class. We repeated the Wilcoxon summed-rank test used in the supervised analysis, after removing from consideration the 500 probes used in clustering. Comparing the serous-type cluster with the control-type cluster, 35 probes remained significant at p<0.01 after stringent Bonferroni correction for multiple tests and 60 remained at p<0.05 (Table 3). The results showed a mixture of hyper- and hypomethylation relative to the controls (Figure S3). An IPA analysis identified known biomarkers for ovarian cancer among this list (Figures S4 and S5). Two genes with recorded relevance to DNA methylation were identified, including *DNMT3A*, a DNA methyltransferase gene and *RB1*. *APC* (from the beta-catenin pathway), *RBAK1* (an *RB1* interaction partner), *MAPK15* and *MAP2K2* kinases, and histone deacetylase *HDAC1* were also on the list (Table 3). Although the supervised and unsupervised analyses utilized different comparator sets, the gene lists contained 10 overlapping entries (Table 3).

**Table 3:** Differential methylation of genes in cluster-based analysis, p<0.05.

Gene	Chr	Pos	ControlMedian	SerousMedian	P-value	Probe	IPANetwork
*RBAK*	7	5051647	0.27	0.51	5.6e-05	cg06914598	1
*MOS*	8	57188855	0.10	0.34	9.9e-05	cg22411207	1
*GSTP1*	11	67107075	0.81	0.41	0.00016	cg05244766	1
*PLEKHF1* *	19	34847706	0.49	0.24	0.00027	cg05512099	
*MAPK15*	8	144869951	0.74	0.46	0.00027	cg11695358	2
*FLJ22555* *	2	200527315	0.73	0.52	0.00027	cg15761233	
*HTATIP2*	11	20341661	0.53	0.29	0.00027	cg18788940	
*S100A8* *	1	151630113	0.51	0.20	0.00027	cg20070090	2
*AHR*	7	17304501	0.55	0.24	0.00042	cg13676215	1
*NNAT*	20	35583164	0.65	0.86	0.00042	cg23566503	
*IL6*	7	22732680	0.20	0.10	0.00063	cg01770232	2
*LGP1* *	17	37600206	0.22	0.08	0.00063	cg08468689	
*OR10J1*	1	157675831	0.52	0.25	0.00065	cg15700197	
*PDPK1* *	16	2527081	0.77	0.36	0.00094	cg14444710	1
*GPR123*	10	134734167	0.54	0.28	0.00094	cg21607649	2
*CCT6A*	7	56085732	0.56	0.25	0.00094	cg23839680	1
*FLJ00060* *	19	59739533	0.82	0.43	0.0014	cg03602500	
*BTNL2*	6	32482732	0.68	0.32	0.0014	cg25391023	2
*S100A8*	1	151630204	0.43	0.12	0.002	cg24898863	2
*ZNF540*	19	42733963	0.23	0.47	0.002	cg27389185	
*LILRA5*	19	59516087	0.40	0.26	0.0027	cg06392096	
*PARP3* *	3	51951707	0.67	0.39	0.0027	cg12554573	
*MAP2K2*	19	4074852	0.16	0.30	0.0027	cg24748945	1
*AIF1*	6	31691437	0.16	0.34	0.0038	cg21440587	2
*WBP11*	12	14848787	0.65	0.43	0.0038	cg22833175	
*CYP4F11*	19	15906788	0.39	0.18	0.0053	cg03190825	
*FLJ44674*	16	47935998	0.78	0.40	0.0053	cg13897627	
*DPP6*	7	154059965	0.69	0.40	0.0053	cg26738880	
*MGC15523*	17	76884479	0.46	0.22	0.0071	cg00466249	
*PRAME*	22	21231596	0.47	0.27	0.0071	cg05208878	2
*RB1*	13	47793174	0.51	0.65	0.0071	cg19254235	1
*DCAKD*	17	40495243	0.53	0.31	0.0096	cg09214551	
*MRGPRX4* *	11	18149936	0.59	0.33	0.0096	cg16446783	
*KPNA1*	3	123716824	0.15	0.05	0.0096	cg25564800	
*LOC126248*	19	38314931	0.58	0.84	0.0096	cg26687173	
*M-RIP*	17	16886972	0.35	0.53	0.013	cg02889982	
*CACNG3*	16	24173204	0.68	0.40	0.013	cg04721098	
*CBFB* *	16	65620188	0.26	0.10	0.013	cg06766367	
*C1QC*	1	22841927	0.33	0.11	0.013	cg11393848	
*INSL4*	9	5221360	0.81	0.55	0.013	cg19297688	2
*APC*	5	112101585	0.07	0.10	0.013	cg24332422	
*MYH1*	17	10360224	0.74	0.49	0.017	cg00134787	
*LUC7L*	16	220047	0.70	0.49	0.017	cg07080946	
*KRTAP13-4*	21	30724700	0.71	0.47	0.017	cg14062083	
*C18orf37*	18	31332717	0.85	0.76	0.017	cg27318281	
*C11orf38*	11	125154608	0.53	0.30	0.022	cg07747336	
*CCDC47*	17	59203744	0.44	0.18	0.022	cg20131968	
*DNAJC14* *	12	54509148	0.21	0.09	0.029	cg11380624	
*NFS1*	20	33752164	0.90	0.85	0.029	cg14963897	
*DNMT3A*	2	25419299	0.74	0.57	0.029	cg21629895	1
*LOC284739*	20	62139546	0.40	0.25	0.029	cg22940152	
*PTAFR*	1	28375495	0.76	0.60	0.029	cg24354652	2
*ANKMY2*	7	16653296	0.38	0.17	0.037	cg25778479	
*TXNL4A*	18	75850362	0.73	0.51	0.048	cg02955504	
*PCDHGA12*	5	140790321	0.21	0.60	0.048	cg07730329	
*EDG4*	19	19600820	0.34	0.13	0.048	cg10521852	
*STRN3*	14	30566605	0.77	0.46	0.048	cg15301694	1
*DCC*	18	49292066	0.79	0.72	0.048	cg18572014	1
*FGF22*	19	591346	0.60	0.41	0.048	cg22189019	
*UNC5CL*	6	41115500	0.78	0.56	0.048	cg22346765	1

1 = IPA network for Cell Cycle and Cell Morphology, 2 = IPA network for Inflammatory Response.

* appears in supervised and unsupervised lists.

Considering the endometrioid-cluster versus the control cluster, we determined that the number and degree of differentially methylated sites increased by more than an order of magnitude. For example, 954 probes were significant at p<0.01. The sheer number of hypermethylated sites suggests an underlying defect in DNA methylation pathways, and limits the utility of considering altered methylation of individual genes.

### Validation of differential methylation

**Figure 3:**
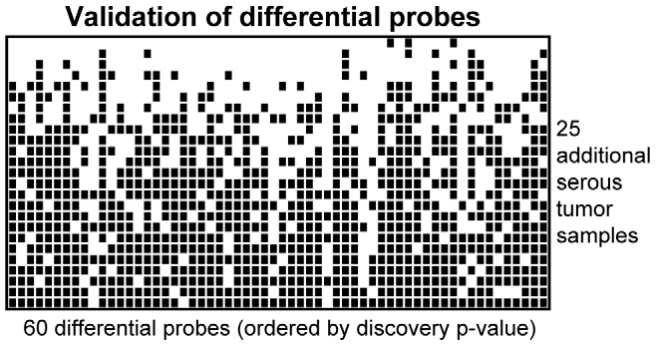
Classification of 25 additional serous tumors. Methylation at 60 loci was used to evaluate an independent set of serous tumors. Each row represents an individual sample; each column corresponds to one of the previously identified differentially methylated sites. An empty box indicates methylation more similar to the control cluster (not necessarily unmethylated); a filled black box indicates methylation more similar to the cluster of ovarian serous tumors. Samples are ordered by the number of sites having methylation resembling the serous tumor group.

To explore whether our set of 60 differentially methylated sites in serous tumors was reproducible, we assessed the methylation of 25 additional samples that were independent of the original analysis set. All were typed as ovarian serous carcinoma and analyzed independently (independent in ascertainment and in time of analysis) from the originals. For each site and for each validation sample, we assessed whether the methylation more closely resembled the normal sample cluster or the serous tumor sample cluster. Of the 25 tumor samples, 4 closely resembled the methylation pattern of normal samples, seven were altered at 21 or more of the 60 loci, and 14 samples showed the altered pattern at 40 or more of the 60 loci (Figure 3).

### Evaluation of published methylation events

Our analysis of differential methylation focused on changes that defined characteristics of each group and were shared among all or nearly all samples. Many important changes in methylation state, previously reported in the literature, have lower prevalence and are not directly identified by our approach. When we assessed our samples for patterns of known methylation, our data were consistent with published results. For instance, *BRCA1* was hypermethylated in 2 of 16 (12.5%) of ovarian serous samples. The tumor suppressor *RASSF1A* showed evidence of complete methylation in 11 tumors, and single-copy methylation in 4 more (31% of serous, and 60% of endometrioid). These changes, and others, are likely to be important transformative events, but are restricted to smaller subsets of the samples.

### Gene ontology & pathway analyses

To tie these results to the literature on ovarian cancer, we performed a gene ontology (GO) analysis and an IPA analysis of the differentially methylated genes from the cluster-based comparison of serous tumors versus controls. The genes corresponding to the methylated loci in our list showed a statistically significant enrichment for GO terms involving regulation of cell cycle (Table 4). The top two networks identified by IPA included “Cell Cycle and Cell Morphology” and “Inflammatory Response” with network scores of 24 and 23, respectively. The network score is based on a hypergeometric distribution and is calculated with the right-tailed Fisher's Exact Test, implying that there is a 1 in 10^23^ or 10^24^ probability of either network occurring from a random list of genes (Table 4, Figure S4,S5). Notably, differentially methylated genes had a large presence in these networks, interacting with important genes in ovarian tumor development including *AKT*, *PI3K*, *VEGF*, and estrogen receptor.

**Table 4:** Gene ontology analysis, genes with differential methylation.

Molecular Function	Gene Count	TotalGenes	P-value	Overlapping Gene set
GO:0005515 – protein binding	29	9005	0.00129	*AHR APC CBFB CCT6A CDC47 DCC DNAJC14 EDG4 FGF22 GSTP1 HTATIP2 IL6 INSL4 KPNA1 LUC7L M-RIP MAP2K2 MAPK15 MYH1 NFS1 PARP3 PCDHGA12 PDPK1 PRAME RB1 S100A8 STRN3 UNC5CL WBP11*
GO:0045786 – negative regulation of progression through cell cycle	5	225	0.00486	*AIFL APC DCC HTATIP2 RB1*
GO:0007049 – cell cycle	8	839	0.00527	*AHR AIFL APC DCC HTATIP2 RB1 STRN3 TXN14A*
GO:0006954 –inflammatory response	6	291	0.0016	*AIFL C1QC CYP4F11 IL6 PTAFR S100A8*
GO:0009611 – response to wounding	6	423	0.00527	*AIFL C1QC CYP4F11 IL6 PTAFR S100A8*
GO:0006950 – response to stress	9	1222	0.0083	*AHR AIFL APC C1QC CYP4F11 IL6 PARP3 PTAFR S100A8*

Uses Benjamini-Hochberg multiple testing correction.

## Discussion

This work represents one of the largest studies of methylation using several normal and tumor subtypes of gynecologic cancers. We initially examined the methylation status of 27,578 sites for 49 samples including normal fallopian tube and endometrium, serous ovarian cancer, endometrioid ovarian cancer, primary endometrioid endometrial cancer, and ovarian metastasis of endometrial cancer. Regardless of tumor or normal status, all samples showed similar profiles in the overall distribution of methylated sites. Although we did not find global shifts toward hyper- or hypomethylation across the assayed samples, a subset of samples showed drastically altered methylation for the X chromosome, consistent with loss of the inactive X chromosome, amplification of the remaining active X, or both [28]. Examples of aneuploidy, including the autosomes, are common in high-grade serous ovarian cancer and are not directly ascertained by this analysis, but influence the proportional methylation levels at each locus. Therefore we removed all probes on the X-chromosome from our dataset.

Our data confirm that different histological subtypes have distinct patterns of methylation. Moreover, ovarian serous tumors are more similar to normal ovarian and endometrial tissues than to ovarian or endometrial endometrioid tumors, which are highly similar to each other and display drastic and consistent changes in their methylation. This result is consistent with a methylator phenotype and in agreement with a model of ovarian endometrioid tumors arising from endometriosis, where the cells ultimately derive from a uterine lineage. Endometrioid tumors from the ovary and uterus share several common somatic mutations [6], and these data support a similar pathogenic mechanism. The marked differences in methylation profiles between histological subtypes underscore the importance of characterizing tumors at the molecular level in order to develop tailored treatment strategies.

For the identification of differentially methylated loci, we used known labels and blinded (data-directed) subgroups. Known labels identified a few dozen genes, some with characterized roles in ovarian cancer. However, given the stringent bar for statistical significance in testing very large numbers of sites, we found that a few outliers within a group could obscure important patterns. By clustering data in an unbiased approach, we found similar methylation patterns among normal samples and some tumor outliers, indicating that current histologic subtyping strategies may miss important molecular distinctions between tumors. This point was further supported in metastatic endometrioid tumors, which also contained an outlier that looked like a normal sample in its methylation patterns. Our clustering approach clearly identified a set of 500 genes that could separate the majority of serous samples from endometrioid samples and normal controls. Although the clustering was distinctive for the three main classes of samples, its use precluded a statistical evaluation of the significance of genes within the set. Nevertheless, the increased power of clustering 49 samples identified an additional 60 loci that were independent of the clustering set and segregated samples into normal or serous subtypes with statistical significance. Several of these genes correspond to networks implicated in the development of ovarian cancer (Figures S4 and S5). We investigated the overlap between gene lists of statistically significant genes identified in the supervised and unsupervised approaches and found 10 genes. Notably, the kinase *PDPK1* is in the PI3K signaling pathway involved in serous ovarian cancer [37]. *PDPK1* and *PLEKHF1* share a pleckstrin homology domain, capable of binding inositol polyphosphates. *PARP3* is involved in DNA repair and genome stability. Given the reproducible signal from these genes regardless of method, we conclude that uncharacterized genes in this list are strongly implicated in ovarian tumor development and require additional characterization.

A limitation of our analysis is that we did not screen the tumor DNA for gene mutations or ascertain gene expression levels; nevertheless, we found that *RB1* and *RBAK* are differentially methylated between the papillary serous and normal fallopian tube samples. *RB1* was recently reported by TCGA to be involved in serous tumor etiology, through mutation or deletion in 67% of tumors [38]. The involvement of the *RB1* pathway is consistent with concurrent Rb1 and Tp53 mutation in mice, which simulates characteristics of aggressive serous ovarian cancers, including formation of ascites and metastasis [39]. Although we did not find significant overlap with the list of methylated genes in serous tumors published by TCGA, this discordance may be due to methodological issues. For example, we do not limit the gene list to candidates that become hypermethylated and found many that lose methylation. Furthermore, we required that scoring be consistent among all tumors. TCGA limits scoring to the top 10% of tumors. Additionally, we did not limit results to genes that become silenced, as methylation has been shown to cause both positive and negative regulatory outcomes [40].

Our analysis of methylation profiles in ovarian and endometrial tumors indicates value in characterizing tumors at the molecular level. The methylator phenotype indicates an aberration in the molecular function of enzymes regulating DNA methylation levels and suggests that a molecule acting upstream of the candidate genes is responsible for the cascade of events leading to tumor development. Studies in hepatocellular carcinoma have identified mutations in the beta-catenin gene in association with a methylator phenotype. Mutations in beta-catenin are also common in endometrial tumors [1], and suggest follow-up experiments to assess a direct relationship to DNA methylation in endometrioid tumors. Moreover, therapeutic strategies aimed at preventing extensive methylation (such as 5-aza-2′-deoxycytidine) should be evaluated in the context of tumors with a methylator phenotype.

The consistency of the methylation profiles, despite independent sample preparation and data collection for TCGA samples, was used to validate and extend our results. These data show that sample batch effects are minimal and do not disrupt data consistency. Our data provide a foundation for future genomic and genetic analyses of endometrial and serous tumors for diagnostic and treatment applications. Notably, our results show that methylation levels in serous tumors are less consistent than endometrioid tumors, but increase and decrease in a target-dependent way. In contrast endometrioid tumors show extensive changes that are likely linked to a common upstream mechanism gone awry.

## Materials and Methods

### Sample collection

Ovarian, endometrial and fallopian tube tissues were received from the Magee-Womens Hospital Tissue Procurement Program (Pittsburgh, PA). The tissues were snap frozen after surgery and stored at −80°C. Genomic DNA was isolated using the Puregene Blood Kit (Qiagen) following the manufacturer's instructions. DNA quality was assessed using a SmartSpec Plus spectrophotometer (BioRad, Hercules, CA).

### Endometrial normal samples

Tissue samples were provided by the Cooperative Human Tissue Network, which is funded by the National Cancer Institute. Samples are from post-menopausal individuals with atrophic endometrium and were obtained from routine hysterectomy or pelvic resection for non-endometrial cancers. DNA was isolated following the protocol of Trizol reagent (Invitrogen).

The use of human subject material was approved by the University of Pittsburgh and the Office of Human Subjects Research at the NIH.

### TCGA data

TCGA data were downloaded at the time of this analysis from the data portal (http://tcga-data.nci.nih.gov/tcga/dataAccessMatrix.htm). Data from the same batch contained unmatched serous tumors and normal fallopian tube samples. Normals: TCGA-01-0639-11A-01D-0383-05, TCGA-01-0631-11A-01D-0383-05, TCGA-01-0642-11A-02D-0383-05, TCGA-01-0628-11A-01D-0383-05, TCGA-01-0637-11A-01D-0383-05, TCGA-01-0633-11A-01D-0383-05, TCGA-01-0630-11A-01D-0383-05, TCGA-01-0636-11A-01D-0383-05. Tumors: TCGA-09-0367-01A-01D-0359-05, TCGA-09-0365-01A-02D-0359-05, TCGA-09-0366-01A-01D-0359-05, TCGA-09-0369-01A-01D-0359-05.

### DNA preparation

DNA was treated with bisulfite according to the protocol of Zymo Research (Irvine, CA), with slight modification. One half microgram of DNA was used for each conversion reaction. The hybridization reaction was performed according to the HumanMethylation27 Illumina BeadChip protocol and scanned using an Illumina iScan System.

### Methylation analysis

Experimental confidence levels were recorded as p-value estimates for each methylation ratio measurement; all readings with a corresponding p-value >0.05 were censored. These low-confidence values were not uniformly distributed in the data, therefore a few loci had an unusually large number of exclusions; we chose to completely eliminate from consideration any probe location at which values for ten or more samples were unavailable. This step eliminated 61 loci.

### Supervised analysis

We performed a comparison between normal fallopian tube (control) and ovarian serous tumors. We excluded probes with poor quality control metrics from the Illumina analysis software (61 probes), and probes that had high variability within the control samples (those with variance in the top 5%, 1,379 probes). We also censored all data from the X and Y chromosomes (1,092 probes). Some overlap in these sets resulted in eliminating a total of 2,489 loci. Differential analysis by Wilcoxon summed-rank test was performed with the R function wilcox.test, followed by Bonferroni correction for the 25,102 loci tested.

### Clustering

Unsupervised clustering was performed in R. To select a subset of loci to use, all primary samples were pooled, and the loci were ranked by sample variance. The number of probes considered was determined empirically, based on bootstrap support for clustering results obtained for data sets of 50, 100, 250, or 500 probes. Based on apparent stability of results with 250 or more probes, the top 500 probes were used for all clustering analyses. Model-based top-down clustering was conducted with the β-mixture model described in Houseman, 2008 [33]. K-means analysis was done with the kmeans function, using within-group sum-of-squares to select the number of clusters. Hierarchical clustering analysis used the pvclust package, with the average and complete linkage methods, and the Euclidean, Manhattan, and correlation distance metrics.

To create a classifier from the clustering results, a beta distribution was estimated for each of the 500 loci used for clustering. These distributions were estimated separately for each of four groups of samples: the endometrioid-type tumor cluster, the serous-type tumor cluster, the control-type cluster, and a cluster including both the control-type and serous-type tumor samples. These groupings allowed a nested binary decision first between endometrioid-type and all other samples, and then a second division between the serous tumor and control clusters. The test metric was calculated as the log of the ratio of probability densities at the observed methylation level for the new sample, summed over all 500 loci.

### Differential analysis

In a pairwise comparison of a tumor cluster versus the control-type cluster, we selected cases showing consistent signal in the controls and alteration in the tumors. We again excluded probes with poor quality, high variability in controls, and sex chromosome location; we also excluded all probes used in clustering for the definition of classes (total: 2812). Differential analysis was again done with Wilcoxon summed-rank test, correction for 24,766 independent tests.

### Validation of differential methylation

For each probe in the set of differentially methylated sites, a threshold was chosen that maximized the discrimination between the previously identified control and serous clusters, minimizing the total number of classification errors (false positives and false negatives). 25 additional samples from ovarian serous tumors were analyzed for DNA methylation as described above, and were assessed for which group they were classified with at each of the differential sites.

## Supporting Information

Figure S1 **Comparison of methylation intensity plots from independent replicates and samples.** Technical replicates of methylation signals in normal fallopian tube, endometrial tumors or ovarian metastases from primary endometrial samples, with best linear fit.
(TIF)

Figure S2 **Clustering results for hierarchical and non-hierarchical methods.** At top, the tree shows the result of top-down partitioning under a beta-mixture model. Below, the six trees show results of hierarchical clustering under either complete linkage (middle row) or average linkage (bottom row), for each of 3 distance metrics. For each method, color bocks beneath the tree show the correspondence to the consensus clusters, with the control-type cluster in blue, the serous-type cluster in black, and the endometrioid cluster in red. For hierarchical methods, black dots on tree nodes indicate ≥95% confidence in that grouping under bootstrap analysis.
(EPS)

Figure S3 **Box plot graph of differentially methylated loci.** Each differentially methylated gene, listed from top to bottom by p-value is represented by a box plot for the methylation in the control cluster (black) and the methylation in the serous tumor cluster (red).
(EPS)

Figure S4 **IPA network for Cell Cycle and Cell Morphology.** Differentially methylated genes participating in the network are colored red with increasing intensity representing smaller p-values. Shapes of molecules indicate distinct molecular functions. Arrows represent direct and indirect interactions. Designations for biomarkers are highlighted in green.
(TIFF)

Figure S5 **IPA network for Inflammatory Response.** Differentially methylated genes participating in the network are colored red with increasing intensity representing smaller p-values. Shapes of molecules indicate distinct molecular functions. Arrows represent direct and indirect interactions. Designations for biomarkers are highlighted in green.
(TIFF)
