# Supplementary figures and images for: Differential Analysis of Ovarian and Endometrial Cancers Identifies a Methylator Phenotype
Source: PLoS One. 2012 Mar 5;7(3):e32941. doi: 10.1371/journal.pone.0032941 (PMC3293923; doi:10.1371/journal.pone.0032941)

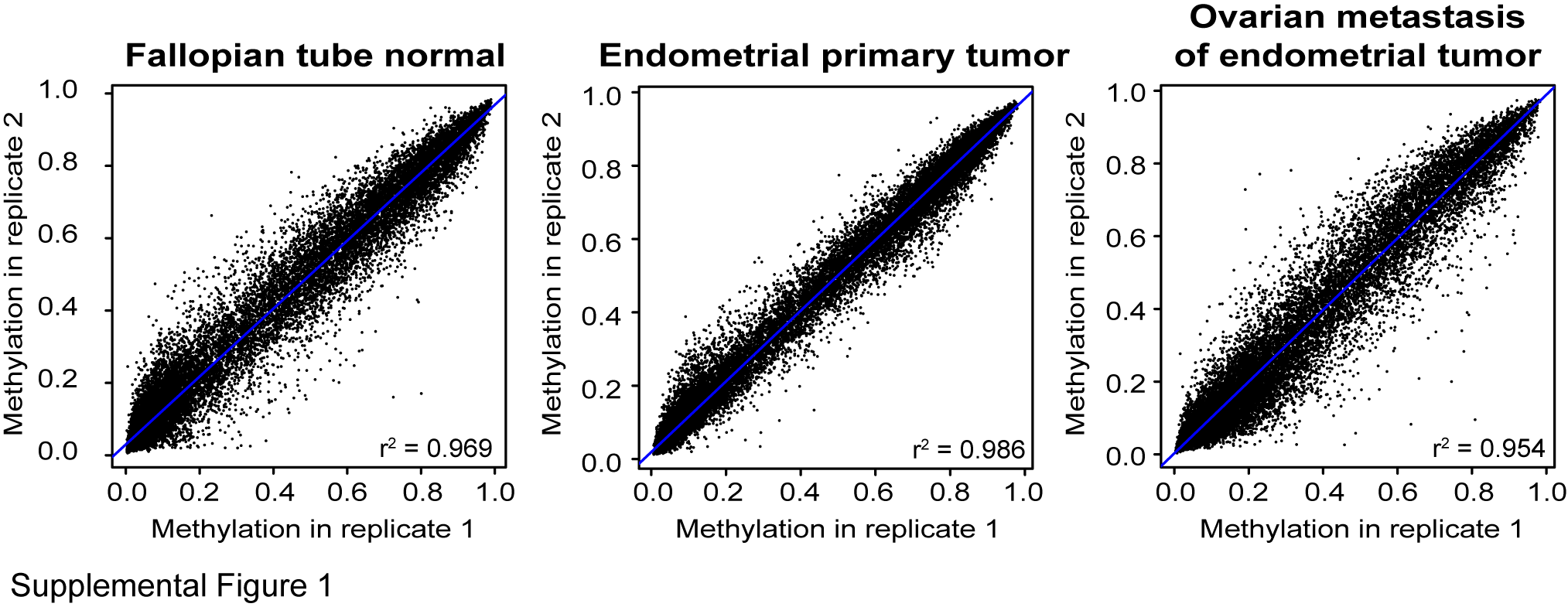

Supplement: Figure S1 — Comparison of methylation intensity plots from independent replicates and samples. Technical replicates of methylation signals in normal fallopian tube, endometrial tumors or ovarian metastases from primary endometrial samples, with best linear fit. (TIF) [file pone.0032941.s001.tif]

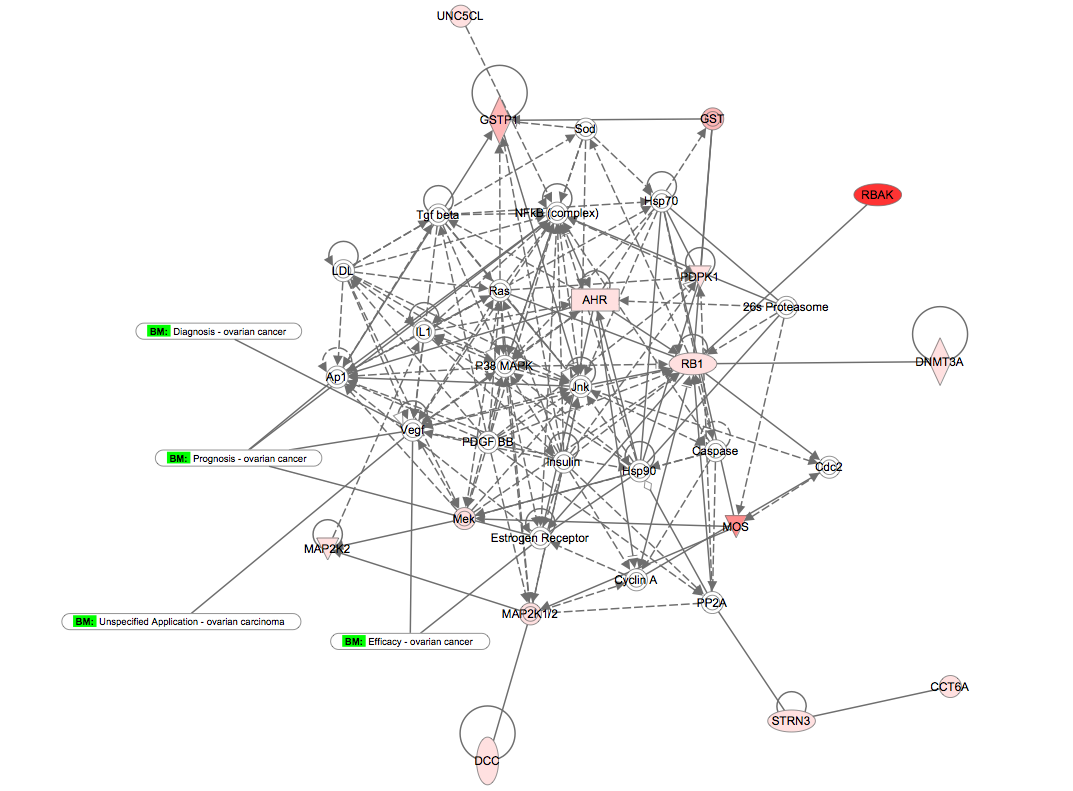

Supplement: Figure S4 — IPA network for Cell Cycle and Cell Morphology. Differentially methylated genes participating in the network are colored red with increasing intensity representing smaller p-values. Shapes of molecules indicate distinct molecular functions. Arrows represent direct and indirect interactions. Designations for biomarkers are highlighted in green. (TIFF) [file pone.0032941.s004.tiff]

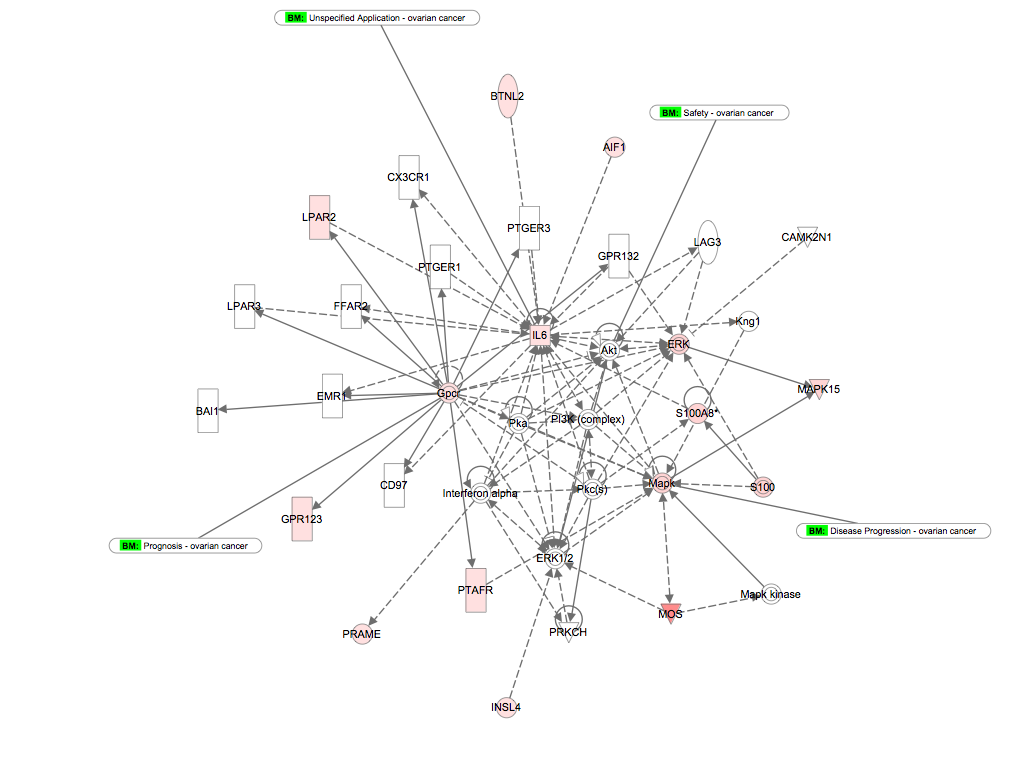

Supplement: Figure S5 — IPA network for Inflammatory Response. Differentially methylated genes participating in the network are colored red with increasing intensity representing smaller p-values. Shapes of molecules indicate distinct molecular functions. Arrows represent direct and indirect interactions. Designations for biomarkers are highlighted in green. (TIFF) [file pone.0032941.s005.tiff]
